# Phase-Field Modeling of Fused Silica Cone-Crack Vickers Indentation

**DOI:** 10.3390/nano12142356

**Published:** 2022-07-09

**Authors:** Zoran Tomić, Krešimir Jukić, Tomislav Jarak, Tamara Aleksandrov Fabijanić, Zdenko Tonković

**Affiliations:** 1 Faculty of Mechanical Engineering and Naval Architecture, University of Zagreb, Ivana Lučića 5, 10000 Zagreb, Croatia; kresimir.jukic@fsb.hr (K.J.); tomislav.jarak@fsb.hr (T.J.); tamara.aleksandrov@fsb.hr (T.A.F.); zdenko.tonkovic@fsb.hr (Z.T.); 2 ITAP, School of Industrial Engineering, University of Valladolid, Paseo de Cauce 59, 47011 Valladolid, Spain

**Keywords:** phase-field modeling, fused silica, Vickers indentation, cone crack

## Abstract

In this paper, a 3D phase-field model for brittle fracture is applied for analyzing the complex fracture patterns appearing during the Vickers indentation of fused silica. Although recent phase-field models for the fracture caused by the indentation loading have been verified by some simpler academic axis-symmetric examples, a proper validation of such models is still missing. In addition, heavy computational costs, and a complicated compression stress field under the indenter, which demands different energy decompositions, have been identified as the most important impediments for the successful application of the phase-field method for such problems. An adaptive strategy is utilized for reducing the computational costs, and some modifications are introduced, which enable an accurate simulation of the Vickers indentation fracture. Here, the fracture initiation ring outside the contact zone is detected by using different energy decompositions, and the dominant cone-crack formation under the Vickers indenter is observed. Different contact conditions are investigated. The proposed model is validated by experimental measurements, and a quantitative and qualitative comparison between experimental and numerical results is conducted.

## 1. Introduction

In recent years, the instrumented indentation test has gained massive popularity to determine the mechanical behavior of different materials due to its straightforward and standardized experimental procedure. The method requires only a small volume of material for measurement, minimal specimen preparation, and low costs, at the same time enabling the evaluation of the indentation by considering both the force and the displacement during plastic and elastic deformation [1]. By monitoring the complete loading cycle of increasing and removing the test force, a wide range of mechanical properties, such as the Martens hardness (*M*s), the indentation hardness (*H*_IT_), the indentation modulus (*E*_IT_), the plane strain modulus (E*), the indentation creep (*C*_IT_), the indentation relaxation (*R*_IT_), and the elasto-plastic behavior (work, *W*), can be determined in one measurement [1]. Based on the measurement results, it is possible to construct stress–strain diagrams, which are of great importance for materials and coatings for which the conventional static tensile test is not applicable. In addition to the above-mentioned mechanical properties, by applying higher loads, it is possible to estimate the indentation fracture toughness of the material based on the cracks at the tips of the Vickers indent.

These mechanical quantities are calculated based on the indenter’s contact (projected) area. Crucial differences can arise when comparing the actual contact area with the area calculated assuming an ideal indenter geometry, particularly at small, measured indentation depths. These differences occur due to the rounding and wear of the indenter’s tip following use. For that reason, it is necessary to determine the actual indenter’s contact surface (area) and use it to calculate the material parameters, ensuring the accuracy and repeatability of the measured values. An indirect way to determine the actual contact area is periodical calibration by utilizing indentations into a material of certified indentation modulus and Poisson’s ratio, known as the reference material [1].

The reference materials known as the reference blocks are specially produced to assure the structure’s necessary homogeneity, uniformity, and long-term stability. In that way, the homogeneity of mechanical properties is confirmed. Stable temperature and humidity properties, as well as an amorphous molecular structure and an almost pure homogenous microstructure, established the position of the fused silica glass as the most investigated/most used reference material for instrumented indentation tests. Since it has a relatively low modulus of elasticity and Poisson’s ratio, fused silica is supremely brittle. According to the calibration certificate, it is not recommended to perform indentations over 100 mN using the Berkovich or Vickers indenter due to cracks that may influence the results. During this research, the indentation forces were increased to initiate a crack on the corner of Vickers indentations.

The crack shapes appearing in brittle materials are related to the indenter shape and to the nature of the material [2]. According to Lee et al. [3], there are five major crack types formed after unloading the sample: radial, lateral, median, half-penny, and Hertzian cone cracks [2]. Independently of the indenter’s geometry (sharp Vickers or Berkovich, spherical, flat punch), similar crack patterns can be observed in fused silica, with the preferential mode being a cone crack or other types of median-radial cracking [4]. The initiation of the cone crack is formed on the specimen surface outside the contact zone of the indenter and the fused silica specimen. The propagation of the cone crack has the incline angle, α, in a range from 30°, as reported by Hagan [5], to 45°, as reported by Michel et al. [6]. In addition, it has also been established that the incline angle depends mainly on the Poisson’s ratio [7].

Hagan [5] concluded that the cone-crack formation is driven only by the elastic stress during the indentation, and the initiation ring is formed only due to positive (tensile) radial stress outside the contact region [8]. One of the indentation fracture theories presented by Lawn and Evans [9] indicates that medial (radial, half-penny) cracks are driven by the stress nucleation caused by material flaws under the indenter, and that a flaw has to have a finite size to initiate a median crack. These conclusions are compared with our numerical modeling in the following sections.

Currently, for the modeling of the indentation crack nucleation and propagation, the most used numerical approach is the cohesive zone modeling (CZM). Bruns et al. [2] modeled the cracking of fused silica under the Berkovich indenter. The authors proposed using the Drucker-Prager Cap plasticity model, which is able to reproduce elasto-plastic material behavior and the hardening due to material densification. Lee et al. [3] also used CZM to analyze the cracking of a brittle material under a four-sided pyramid (Vickers indenter). The biggest drawback of CZM is that the crack nucleation and propagation directions have to be determined a priori: cracking appears only in the a priori defined cohesive planes. This makes CZM suitable only for capturing the radial or half-penny cracks, but not the cone cracks, which are not planar. Among other possible approaches, the extended finite element method (XFEM) [10] and the peridynamics [11] have been attempted. Although both methods are capable of reproducing complex crack patterns, their usage in 3D modeling is still a daunting task, because they tend to become extremely complex due to problems associated with the crack tracking in XFEM, or a proper definition of internal forces and efficient numerical implementation in peridynamics.

The phase-field (PF) method seems to be an appropriate approach to overcome the latter problems. It does not require ad hoc criteria for the nucleation, propagation, or branching of cracks, and its numerical implementation in the existing FEM codes is relatively simple and straightforward. However, due to high computational costs, mainly caused by the need to use very dense meshes in the regions where damage appears, only 2D models have so far been proposed. In general, it has been shown that one of the main problems in phase-field modeling of the indentation process is accurate modeling of crack initiation in an otherwise defect-free material. Additional considerable problems include the proper definition of the length scale parameter value or the definition of the crack driving force. Strobl and Seeling [8] proposed a 2D phase-field brittle formulation for the simulation of cone-crack formation under the flat indenter. They have studied various modifications of standard AT1 and AT2 models, which render the phase-field method more suitable for indentation simulations. In doing so, they identified the most important problems and proposed certain conditions that have to be fulfilled by the brittle phase-field models in indentation simulations, such as: using appropriate energy decompositions and crack driving functions to account for the tension-compression behavior and crack boundary conditions, using small length scale parameter values in comparison to characteristic crack dimensions to ensure the accurate capture of crack initiation, defining appropriate degradation functions that prevent erroneous phase-field evolution and ensure the linear response before the initiation of damage, and incorporating the material’s tensile strength and fracture toughness as independent material parameters. In their work, they utilized a hybrid approach, where no split is used for the Cauchy stress tensor because the cracks remain opened during the indentation, while the directional split proposed by Steinke and Kaliske [12] is used for the crack driving function to avoid the crack evolution being driven by a compression stress state. Thereby, the crack driving function itself is formulated by using positive principal stresses as it is deemed more appropriate than by using strains. Small values of length parameters had to be used to capture the spontaneous crack initiation, leading to restrictions on the minimal values of the tension strength in their AT2 model. Fourth-order degradation functions were used to ensure the linear elastic response prior to the onset of damage, but such choice compromises the crack propagation to the fully broken state. The overestimation of the surface crack energy, caused by an over-smearing of the phase-field if a relatively large value of length parameter is used, is avoided by a recalibration of the length parameter and yield functions. The irreversibility of the phase-field is ensured by employing the “crack-like” constraint, allowing for irreversibility of the damage before the onset of a well-defined crack. However, such approach does not work well for all simulation setups. Despite all mentioned restrictions, for a certain choice of input variables for which all required conditions are satisfied, their modified model was able to reproduce the main features of cone-crack initiation and propagation: the formation of the initiation ring around the indenter, a vertical crack propagation, and the conical crack propagation with an inclination to the sample surface. Kindrachuk and Klunker [13] used a similar brittle phase-field formulation for the axis-symmetric 2D simulation of spherical indentation to investigate a kinked cone-crack propagation, caused by the changing contact conditions between the propagating spherical indenter and the sample surface. They performed a frictionless contact analysis by employing an ad-hoc crack driving force based on the strain measure, similar to the Beltrami criterion of failure, instead of a stress-based crack driving force. The irreversibility was enforced in a “damage-like” manner by employing a history variable. Only one problem setup was analyzed, and the applicability of the method was not studied in detail. In a very recent work, Wu et al. [14] used an axis-symmetric phase-field cohesive zone model to investigate the flat punch indentation of a homogeneous material with borosilicate glass properties. Therein, the cohesive zone model has been applied to correctly capture the crack nucleation in the otherwise defect-free material. Here, the failure strength can be chosen independently from the fracture toughness by applying the Wu-Nguyen phase-field model for quasi-brittle fracture, where the length parameter is introduced as an independent parameter. This allows for the choice of the length parameter value that is small enough to accurately capture the crack initiation and to prevent the unphysical widening of the damaged zone during the crack propagation. That approach shows impressive potential for correctly capturing all important details of the cracking process caused by the indentation, including the initiation. However, its implementation is more complex than the phase-field models based on the AT2 model, as it requires special solvers for the imposition of the irreversibility condition on the phase-field.

In comparison to the similar phase-field models for the indentation problem, encountered in the available literature, the novelties of the present work are:

- To the authors’ knowledge, the present paper represents the first 3D phase-field model of the Vickers indentation fracture to this date.
- An asymmetric model, where the energy decomposition is applied for both the crack driving force and the stress field, is used.
- Contact analysis with friction is applied.

The proposed numerical model is validated by experimental indentation, showing that the formation of a cone crack is accurately predicted by the current model in the Vickers indentation.

The paper is organized as follows. The experimental setup is presented in Section 2. Afterwards, the most important details of the used phase-field formulation are exposed, along with the energy decomposition, which has a significant influence on the indentation modeling. Then, the numerical model is described in detail. In Section 3, the obtained numerical results are compared with the experimental data. Finally, some conclusions are presented in Section 4.

## 2. Materials and Methods

### 2.1. Material and Experimental Setup

The experimental instrumented indentation test was conducted on a certified reference material—a round fused silica block (ϕ 25 mm×5 mm), produced and certified by Anton Paar, TriTec SA, (Corcelles-Cormondrèche, Switzerland) calibrated by the Berkovich indenter. The measurements were performed on the Micro Combi Tester (MCT^3^) produced by Anton Paar, TriTec SA, (Corcelles-Cormondrèche, Switzerland) according to EN ISO 14577-1:2016, at room temperature using a certified Vickers diamond pyramidal indenter. The measurements were performed using different loads. The load of 50 mN was selected to determine the plane strain modulus, E*, and the indentation modulus, *E*_IT_, in accordance with recommendations from the calibration certificate not to perform indentations over 100 mN using the Berkovich or the Vickers indenter. Higher loads could cause cracks that may influence the results. Based on the measurement, it was concluded that the measured values of E* are in excellent correlation with certified values. The certified and the measured E*, the certified Poisson’s ratio, *ν*, and the fracture toughness value, *K*_IC_, from the literature, used for further phase-field modeling, are shown in Table 1.

The fracture toughness, *K*_IC_, could not be directly measured by the instrumented indentation test, but it could be calculated by measuring the total length of cracks emanating from the corners of indentations at loads higher then critical force, *P*c, at which the first cracks occur. The model used for the calculation of *K*_IC_ is dependent on the type of crack, median, radial, half-penny, cone, or lateral, using the Palmquist method [15] or the method proposed by Anstis et al. [16]. As mentioned in the Introduction Section, during indentation, the load was increased to determine the critical force, to pinpoint the start of crack formation, and possibly, to calculate *K*_IC_.

**Figure 1:**
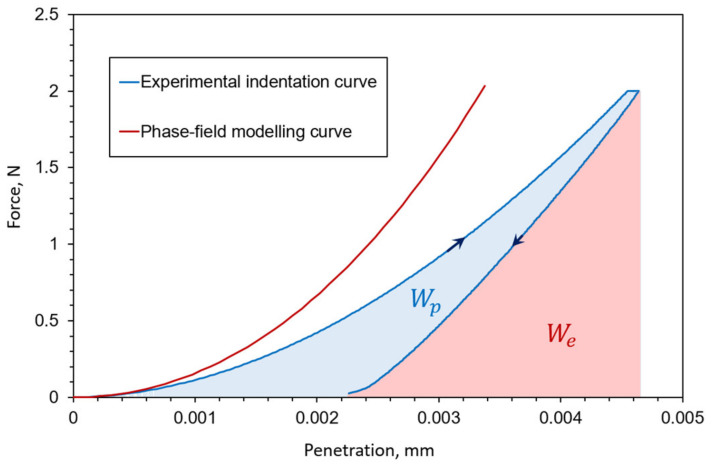
Indentation curve comparison between numerical brittle phase-field formulation and experimental measurement. Area under the experimental indentation curve corresponds to elastic ($W_{e}$) and plastic ($W_{p}$) indentation work.

Such behavior starts to become clearly visible when applying a force of 2000 mN. Finally, the instrumented indentation test was conducted with a load control loop and 2000 mN of force, with the loading and unloading speed of 4000 mN/min and the hold on maximum load for 30 s. The average indentation depth was around 4 µm, and the mean indentation curve is shown in Figure 1 with the displacement resolution of 30 nm.

**Figure 2:**
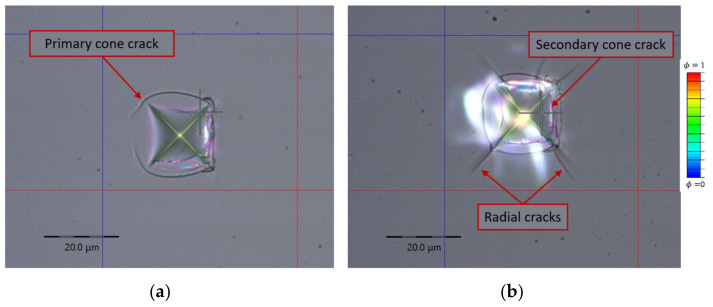
Residual indents and different fracture patterns after Vickers indentation on fused silica glass, (**a**) indent showing only primary cone crack, (**b**) indent with secondary cone crack and radial cracks at the edge of the indent.

Around 15 indentations were conducted, and similar cone-crack patterns were observed (Figure 2). The indentations were optically analyzed on a video microscope and residual indents are presented in Figure 2.

The yield strength, $\sigma_{y}$, of fused silica cannot be directly measured from the indentation measurements. Even though different methods for extraction of elasto-plastic parameters from indentation do exist, such as the one for the low-hardening materials proposed by Giannakopoulos et al. [17] and later upgraded by Dao et al. [18], the yield strength of the fused silica is taken from the literature. Bruns et al. [4] concluded from the values obtained by various measurements that the $\sigma_{y}$ value of fused silica is in the interval from 4000 to 7000 MPa.

As it will be later explained in detail, the phase-field method is very mesh-dependent, at least with the presented formulation, and mesh dependance is linked to material parameters. This means that the characteristic element length, *l*, is a function of material properties, $l=f\left(E,\nu ,\sigma_{max}\right)$. Since fused silica is a brittle material, the value of ultimate strength can be taken as the same as the yield strength. For the purposes of this research, $\sigma_{max}$ has the value of 4000 MPa.

### 2.2. Numerical Modeling

#### 2.2.1. Phase-Field Formulation

Herein, the brittle phase-field formulation is used [19]. The phase-field approach regularizes the sharp discontinuity with a scalar parameter ϕ, which has the value from 0 to 1, distinguishing between the fractured state (ϕ=1) and the intact state (ϕ=0). A strain energy degradation function is introduced (in this case a simple quadratic function, Equation (5)) to account for the loss of stiffness due to fracture propagation. In that case, in regions where ϕ=1, the stress and stiffness drop to zero. For more details about the phase-field method, see [20,21,22]. The basic equations of the applied phase-field formulation are presented in Table 2. Here, Ψ is the energy functional of the body occupying a volume, Ω, containing a crack, with Γ as a crack surface. According to Equation (1), the total energy, Ψ, consists of the stored energy, $\Psi^{b}$, due to deformation and the energy dissipated in the fracture process, $\Psi^{s}$. Therein, $\psi_{e}$ is the elastic strain-energy density function (Equation (2)), and $G_{c}$ is the fracture toughness or the critical energy release rate denotes the small strain tensor, defined as the symmetric part of the displacement gradient, $\varepsilon =\mathrm{sym}\left(\nabla u\right)$, with u as the displacement field. According to the phase-field method for fracture [23], the functional Ψ can be regularized as in Equation (3), where *l* is the length scale parameter, which is here chosen to be a material parameter and can be calculated by Equation (7), according to [24]. In Equation (4), F^ext^ represents the vector of external forces. The history field, *H*(*t*), in Equation (4) is employed instead of $\psi_{e}$ to prevent the crack “healing”.

Although in [5,11] the usage of stress-based energy splits is advocated in phase-field numerical simulations, herein, three different strain-based energy splits are tested: two spectral splits, one proposed by Miehe et al. [20] and the other by Freddi [26], and a volumetric-deviatoric split, proposed by Amor et al. [27].

#### 2.2.2. Energy Split

One of the major aspects of the phase-field modeling of fracture is the choice of energy split. It determines the mechanical behavior of the damaged material and enables the simulation of crack closure. Additionally, the energy split determines the way in which the crack grows, as the crack driving force is determined by the energy split. It is important to emphasize that in real situations, cracks cannot initiate under compressive stress. In its nature, the material during indentation is predominantly in a compressive stress state. From this aspect, the use of an appropriate energy split is the most important part in the process of indentation modeling with the phase-field method.

There exist three basic families of energy splits: the well-established spectral energy splits, spherical-deviatoric energy splits, and the more recently developed directional splits. As noticed in [5,11], in the phase-field indentation simulations, defining a proper energy split is essential for defining the crack driving force to prevent the crack evolution due to compressive stress. On the other hand, so far, no split has been applied for computing the stresses, apparently because in the axis-symmetric cases, such as spherical or flat punch indentation, cracks remain open once they appear. However, for a more general purpose, employing some convenient energy split would be more than beneficial, see, e.g., [9,28,29]. The directional splits are the most promising tools in resolving the problem of crack closure, as the knowledge about crack direction is necessary for its description. Since such splits are relatively new and are still the object of intense research, they are not considered in this work.

Among spherical-deviatoric splits, the most popular one is Amor’s split [4], with positive and negative energies defined as:

(8) $$\psi_{0}^{+}=\frac{1}{2}\left(\lambda +\frac{2}{3}\mu \right)\langle \mathrm{tr}{\left(\varepsilon \right)\rangle }_{+}^{2}+\mu \varepsilon_{D}:\varepsilon_{D},\;\psi_{0}^{-}=\frac{1}{2}\left(\lambda +\frac{2}{3}\mu \right)\langle \mathrm{tr}{\left(\varepsilon \right)\rangle }_{-}^{2},$$

where $x_{\pm }=\frac{1}{2}\left(x+\left|x\right|\right)$ are the Macaulay brackets, *λ* and *μ* are the Lame’s constants, while $\varepsilon_{D}$ stands for the deviatoric part of the strain tensor. Despite its popularity, this split has some serious flaws. If a simple uniaxial compressive case is considered, the nonzero deviatoric part of the strain tensor leads to cracking with a fixed compressive-tensile strength ratio. Additionally, nondegraded energy due to the negative strain tensor trace should lead to the transmission of compressive stresses if the specimen is fully broken. However, in most cases, this split cannot transmit any compressive stresses, because for the fully damaged material it does not take the deviatoric strain into consideration, and therefore the material starts to act as a fluid. For those reasons, such split is not a good choice for the simulation of indentation, where the expected contact-induced compressive stresses are high and lead to cracking in the contact zone and to the fluid-like behavior of the material.

Spectral splits utilize a spectral decomposition of the strain/stress tensor to define the positive strain energy. Some notable spectral splits have been proposed by Miehe [20], Freddi [26], Lo [30] (a 3D generalization of the Freddi split), He [31] (the ‘orthogonal’ split implemented in the phase-field framework by Nguyen [32]), or Wu and Nguyen [33]. In the highly popular split by Miehe, energies are computed as:

(9) $$\psi_{0}^{\pm }=\frac{1}{2}\lambda \langle \mathrm{tr}{\left(\varepsilon \right)\rangle }_{\pm }^{2}+\mu \mathrm{tr}\left(\varepsilon_{\pm }^{2}\right),$$

with positive and negative strain tensor parts, $\varepsilon_{\pm }$, defined as:

(10) $$\varepsilon_{\pm }=\overset{3}{\underset{i=1}{\sum }}{\langle \varepsilon_{i}\rangle }_{\pm }n_{i}⊗n_{i}$$

Herein, $\langle \varepsilon_{i}\rangle {}_{\pm }$ and $n_{i}$ are the positive/negative eigenvalues and corresponding eigenvectors of the strain tensor. Although this split does not degrade energy related to the negative principal strains, it does not completely prevent cracking in compression, which can be caused by the Poisson’s effect. For example, a uniaxial compressive stress state will lead to one negative and two positive principal strains. Those positive principal strains will form a positive strain energy density and will lead to the growth of the phase-field. Consequently, such split is not suitable for contact problems.

In this work, we rely on the spectral split defined by Lo [30]. With the principal strains defined as $\varepsilon_{3}\geq \varepsilon_{2}\geq \varepsilon_{1}$, such split is defined by the equations in Table 3.

However, this set of equations can also be written as:

(15) $$\psi_{0}^{\pm }=\frac{1}{2}\lambda {\left(\mathrm{tr}\left(\varepsilon_{\pm }^{*}\right)\right)}^{2}+\mu \mathrm{tr}\left({\left(\varepsilon_{\pm }^{*}\right)}^{2}\right)$$

where $\varepsilon_{\pm }^{*}$ is defined as:

(16) $$\varepsilon_{\pm }^{*}=\sum_{i}^{3}\varepsilon_{i,\pm }^{*}n_{i}⊗n_{i}$$

where $n_{i}$ are eigenvectors of ε and $\varepsilon_{i}^{*}$ are functions of eigenvalues of ε, defined as:

if $\varepsilon_{1}>0$

(17) $$\varepsilon_{1,+}^{*}=\varepsilon_{1},\;\varepsilon_{2,+}^{*}=\varepsilon_{2},\;\varepsilon_{3,+}^{*}=\varepsilon_{3},\;\varepsilon_{1,-}^{*}=0,\;\varepsilon_{2,-}^{*}=0,\;\varepsilon_{3,-}^{*}=0$$

else if $\varepsilon_{2}+v\varepsilon_{1}>0$

(18) $$\begin{array}{c} \varepsilon_{1,+}^{*}=0,\;\varepsilon_{2,+}^{*}=\varepsilon_{2}+v\varepsilon_{1},\;\varepsilon_{3,+}^{*}=\varepsilon_{3}+v\varepsilon_{1},\\ \varepsilon_{1,-}^{*}=\varepsilon_{1},\;\varepsilon_{2,-}^{*}=-v\varepsilon_{1},\;\varepsilon_{3,-}^{*}=-v\varepsilon_{1} \end{array}$$

else if $\left(1-v\right)\varepsilon_{3}+v\left(\varepsilon_{1}+\varepsilon_{2}\right)>0$

(19) $$\begin{array}{c} \varepsilon_{1,+}^{*}=0,\;\varepsilon_{2,+}^{*}=0,\;\varepsilon_{3,+}^{*}=\varepsilon_{3}+\frac{v}{1-v}\left(\varepsilon_{1}+\varepsilon_{2}\right),\\ \varepsilon_{1,-}^{*}=\varepsilon_{1},\;\varepsilon_{2,-}^{*}=\varepsilon_{2,\;},\;\varepsilon_{3,-}^{*}=-\frac{v}{1-v}\left(\varepsilon_{1}+\varepsilon_{2}\right) \end{array}$$

else

(20) $$\begin{array}{c} \varepsilon_{1,+}^{*}=0,\;\varepsilon_{2,+}^{*}=0,\;\varepsilon_{3,+}^{*}=0,\\ \varepsilon_{1,-}^{*}=\varepsilon_{1},\;\varepsilon_{2,-}^{*}=\varepsilon_{2},\;\varepsilon_{3,-}^{*}=\varepsilon_{3} \end{array}$$

Note that the following two properties always stand:

(21) $$\begin{array}{c} \varepsilon_{+}^{*}+\varepsilon_{-}^{*}=\varepsilon ,\\ \psi_{0}=\left(\;\varepsilon_{+}^{*}+\varepsilon_{-}^{*}\right):C:\left(\;\varepsilon_{+}^{*}+\varepsilon_{-}^{*}\right)=\varepsilon_{+}^{*}:C:\varepsilon_{+}^{*}+\varepsilon_{-}^{*}:C:\varepsilon_{-}^{*},\\ \varepsilon_{+}^{*}:C:\varepsilon_{-}^{*}=\varepsilon_{-}^{*}:C:\varepsilon_{+}^{*}=0, \end{array}$$

where C stands for the elasticity tensor of undamaged isotropic material. While the Miehe’s split degrades energy related to positive principal strains, the Lo’s split degrades energy related to positive principal stresses. Therefore, the Lo’s split does not lead to the growth of cracks in compression. However, like all other spectral splits, it still suffers from the transmission of shear stresses through the crack. However, in our opinion, this split is the most suitable spectral split for problems with contact, such as indentation. As used split of Lo’s is 3D version of spectral split given in work of Freddi, in rest of the work we denote it as Freddi split.

**Figure 3:**
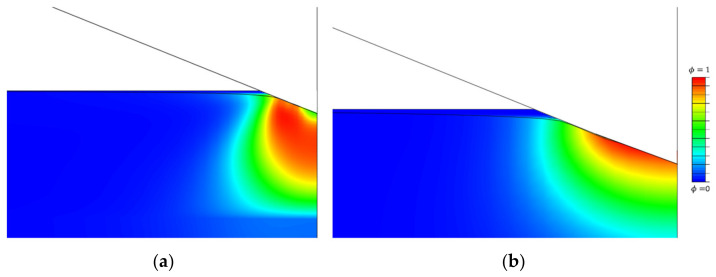
Comparison of (**a**) Miehe and (**b**) Amor energy decomposition.

Figure 3 shows a comparison between the splits by Miehe and Amor. Both energy decompositions fail to mimic the crack propagation under the indenter. In both examples, the scalar phase-field, ϕ, grows under the indenter where the compression stress field occurs. In the case of Amor, a similar growth of damage can be spotted under the indenter, along with the fact that elements lose their integrity and the excessive distortion of individual nodes appears.

The above formulation was implemented in the first-order, eight-node hexahedral finite element into the ABAQUS program package (2020., 2020, Dassault Systèmes, Vélizy-Villacoublayu, France) [34] by following a procedure proposed by Seleš et al. [35], using a three-layered system. In this way, since the second layer is a standard ABAQUS finite element, different interaction features between two (or more) surfaces (or nodes) can be prescribed.

#### 2.2.3. Indentation Modeling

**Figure 4:**
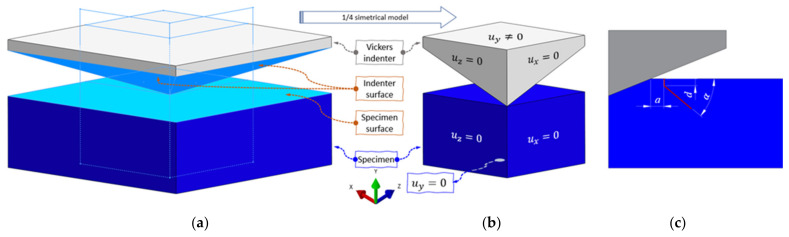
Numerical model of Vickers indentation: (**a**) full model, (**b**) quarter symmetrical model with symmetry boundary conditions, and (**c**) idealized crack.

To reduce the number of degrees of freedom (DOFs), a two-times symmetrical model was used (Figure 4a), with symmetrical boundary conditions defined by Figure 4b. The size of the specimen model is 50 μm×35 μm×50 μm. The loading is prescribed as the displacement as the flat top side of the indenter, which is assumed to be rigid. This assumption has been adopted by different finite element studies [8,13,36], where simulations have exhibited little or no difference between the simulations with a rigid or deformable indenter.

**Figure 5:**
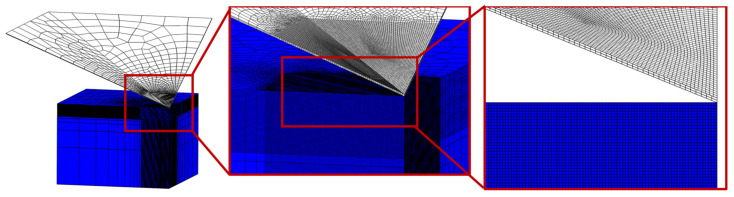
Mesh distribution on the indentation model.

Material parameters for our brittle fracture phase-field formulation are shown in Table 4. The length scale parameter has been calculated according to Equation (7) and has the value of approximately $3\times 10^{-6}$ mm. Elements with an appropriate edge length are placed only below the indenter and in the contact area (Figure 5). The quarter model is discretized by approximately 3.5 M hexahedral elements.

Regarding the contact properties, the indenter surface is taken as the master surface, while a specimen’s penetrated surface is the slave surface. Moreover, the master surface (indenter) has a coarser mesh than the slave surface (Figure 5). The contact behavior is established as a surface-to-surface finite sliding, i.e., the sliding between the master and the slave surface nodes is possible [34]. Furthermore, the contact in the normal direction is presumed as the Lagrange formulation hard contact with a relatively small node penetration, and in the tangential direction as the Lagrange formulation with a friction coefficient.

The applied phase-field model was implemented in the ABAQUS software package (2020, 2020, Dassault Systèmes, Vélizy-Villacoublayu, France) along the lines described in [22]. Herein, presented numerical examples were conducted on a single workstation with the AMD^®^ processor, with a 3.80 GHz clock speed and 128 GB RAM memory. Additionally, ABAQUS was accelerated with a NVidia^®^ RTX™ GPU unit. The average duration of the simulations was around 10 days.

## 3. Results and Discussion

The experimental measurements clearly demonstrate the occurrence of a significant plastic deformation during the Vickers indentation. It is visible as a residual indent after unloading (Figure 2) and the residual plastic energy in Figure 1. Even though the elasto-plastic deformation has been detected by other authors, such as in [2], as an important phenomenon during the Vickers indentation of fused silica, here we applied a brittle phase-field formulation due to the high costs of the phase-field simulations and a complex coupling of damage and plasticity phenomena in the phase-field models. It can be seen from the indentation curve comparison (Figure 1) that the numerical, brittle formulation, can describe the experimental indentation loading curve relatively well, although with a significantly stiffer response caused by the lack of plastic deformation. Therefore, the implementation of a plastic material model will be in the focus of future research since the authors believe that an adaptive elasto-plastic phase-field formulation is a well-suited approach to model the complex Vickers indentation.

Since the presented phase-field formulation is implemented in the commercial FE software ABAQUS, the use of all interaction features is possible. In this model, the contact interaction is prescribed on the contact surfaces, as described in a later section.

The use of other contact parameters did not afford good results, such as small sliding contact formulation or the penalty contact method. Investigation has shown that the indentation curve did not change (quantitatively) in comparison with the final contact modeling (finite sliding and the Lagrange formulation), but the formation of the cracks, i.e., the growth of the scalar phase-field parameter on the surface, did change. Cracks form in the early stages of the loading process and below the indenter itself. The small sliding contact does not allow the motion of the nodes of adjacent surfaces when the contact is established. This means that the slave nodes stick to the master surface as soon as the contact is established. With the progress of the loading process, they stay stuck to the master surface, unlike the finite sliding formulation, which allows the motion of the nodes in the contact.

**Figure 6:**
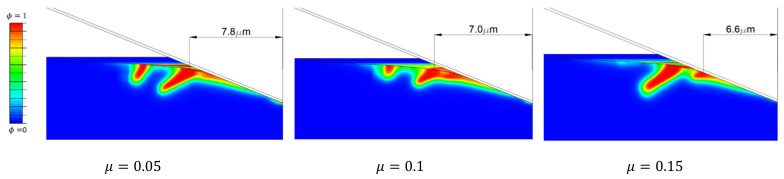
Comparison of the crack growth for different values of the friction coefficient, *µ*. All deformed models are under the same indenter penetration, i.e., h=3 μm.

Friction between the indenter surface and the specimen surface is also an influential parameter (Figure 6). As friction increases, the initial ring forms closer to the indenter centerline. Moreover, the initiation ring of the secondary cone crack (Figure 2b) propagates deeper as friction decreases.

Unlike Strobl and Seelig [8], who concluded that the energy decomposition proposed by Freddi cannot replicate the cone crack under the flat indenter with their 2D phase-field formulation, it was shown here that it is possible to model the cone crack with the Vickers indenter by our 3D formulation employing the above-proposed Freddi decomposition.

At the beginning of the loading phase, as soon as the contact is established, the phase-field starts to rise slowly. Since the Freddi energy decomposition is introduced, the negative compression stress does not influence the crack initiation. Only after the indenter notably penetrates the specimen does the phase-field start to intensely rise. The formation of the damage below the indenter is not noticed, unlike in the model with the Miehe energy decomposition (Figure 3).

**Figure 7:**
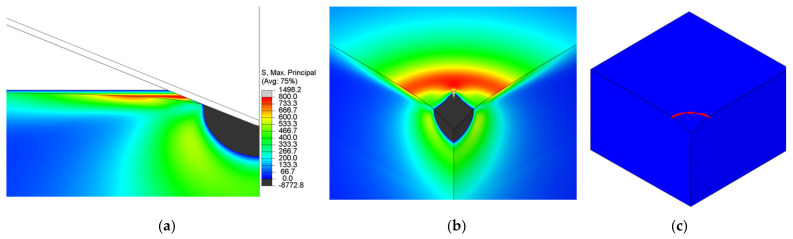
Positive maximum principal stress (in MPa) on specimen surface as a cause of cone-crack initiation, (**a**) stress distribution (in MPa) in front plane, (**b**) stress distribution in isometric view (without the indenter), showing ring-like distribution outside the contact region, and (**c**) isometric view (without the indenter) of the initiation ring.

As reported by Strobl and Seelig [8], the tensile stress on the specimen surface is responsible for the cone-crack initiation. It is visible that the initiation is governed by a weak surface-positive stress field (Figure 7a,b) that forms crack initiation in the shape of a ring. The initiation ring (Figure 7) is located outside the contact region at a certain distance from the indenter contact edge.

By increasing the load, the crack starts to propagate orthogonally to the surface towards the interior of the material (see the dimension, *d*, in Figure 4c). This propagation direction has been well-captured in both 2D numerical [8,13] and 3D experimental [5,6] observations. With increasing load, the crack continues to propagate with an incline angle of around 45° with respect to the surface (Figure 4b).

**Figure 8:**
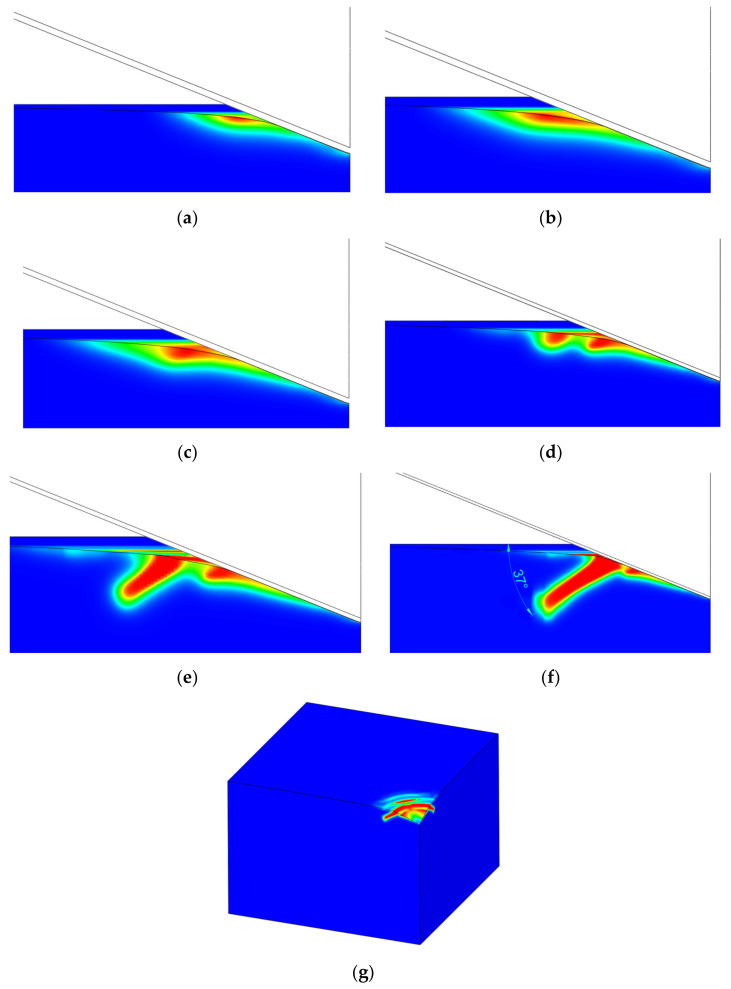
Stable crack growth with inclination angle at different penetration depths: (**a**) u=1.2 μm, (**b**) u=1.65 μm, (**c**) u=2.025 μm, (**d**) u=2.4 μm, (**e**) u=2.8 μm, (**f**) u=3.375 μm, front view for different penetration depths, and (**g**) isometric view without indenter at u=3.375 μm (max. loading).

As reported by recent phase-field modeling by Strobl and Seeling [8] and Wu et al. [14], and an experimental investigation by Kocer and Collins [7], the Poisson’s ratio is the most influential material parameter that influences the angle of the cone crack (in case of a flat punch indenter). However, in the case of an inclined indenter (Vickers or Berkovich), the angle of the cone crack is around 40–45°, as reported by Hagan [5] and Michel et al. [6], which is significantly higher than the angle encountered in the flat punch indentation. In our simulation, a somewhat smaller inclination angle of the cone crack has been observed, as shown in Figure 8f.

The primary and secondary cracks are visible in Figure 8b. The size of the primary (small) cone crack is dependent on the friction coefficient (Figure 6), but both are noticed in experimental measurements (Figure 2), as well as in the relevant literature [37].

Unlike the flat punch, where the contact area is constant, in the Vickers indentation we can expect that the indenter will pass through the crack at some point during the indentation. In terms of the numerical analysis, the crack starts to thicken. This happens when the force is just below 2000 mN. At this force, a new cone crack starts to form, again outside the contact region. Further loading leads to the formation of a new cone crack (Figure 8g), but with a slightly smaller inclination angle.

## 4. Conclusions

As shown in Section 2, from the considered energy splits, only the spectral energy split proposed by Freddi can accurately replicate cone-crack behavior. The decomposition proposed by Amor fails to model the crack since finite elements in the compression state lose their stiffness and excessive distortion of elements occurs. A similar growth of the scalar damage field was observed using the decomposition proposed by Miehe.

As can be seen from the presented results, the phase-field method is capable of modeling the cone crack formation during the Vickers indentation in fused silica if an appropriate energy split is used. The phase-field numerical model can initiate a ring on the specimen surface outside of the contact region of the indenter, as noticed by Hagan [5] and Lawn and Evans [9]. These indentation crack phenomena are also visible in Figure 2, where a cone crack is formed outside the residual indent. This fact is in accordance with the Lawn and Evans theory that says that the cone crack is the dominant crack mode in the indentation of intact fused silica and that radial-median (half-penny) or lateral cracks are formed due to specimen irregularities or flaws beneath the indenter. A crack in the ideal numerical model without flaws is initiated only by a positive stress field on the surface (Figure 7a,b). From the aspect of linear fracture mechanics (assuming brittle materials), fracture occurs, according to the principal stress hypothesis, when the maximum principal stress reaches the fracture strength of a material [4]. This statement can also be corelated with the phase-field formulation and energy splits, where fracture occurs when one positive stress reaches the critical value.

Regarding the contact formulation, as described, the finite sliding contact is the accordance formulation, since small sliding cannot replicate the conditions appearing in the investigated cone crack. The reason for this lies in small sliding formulation, which “glues” the corresponding nodes from the master and slave surfaces. With further penetration, the nodes of the slave surface are dragged by the corresponding master nodes, which creates positive tensile stress on the specimen, i.e., the slave surface.

By further increasing the load, the crack starts to propagate orthogonally to the surface to a certain depth, firstly in the direction parallel to the loading direction and then at a certain incline angle, which corresponds well to the numerical and experimental observations conducted in the relevant literature.

Even though fused silica is usually presented as an elastic, brittle material, from the obtained experimental indentation curve (Figure 1), it is visible that there occurs a significant plastic deformation, also visible after unloading in the form of a residual indent. The authors’ opinion is that no significant change in the crack pattern, with the introduction of a ductile phase-field formulation, will appear since the crack is still driven by only elastic deformation energy.

This research creates new questions in the numerical modeling of indentation cracking, as well as the validation of different phase-field formulations, since fused silica consists of pure silicon dioxide, SiO_2_, and can be assumed to be homogeneous. The use of graphic accelerated servers will enhance the computational speeds in future investigations, especially in combination with efficient adaptive remeshing and faster equation solvers. The use of a ductile formulation instead of a brittle one will possibly describe the real indentation curve even better. Additionally, the use of ductile formulation with different energy decompositions could possibly describe the radial (also median and half-penny) cracks, which appear as the second-dominant crack pattern in the fused silica indentation. This could explain why different crack patterns appear on the same specimen at the same indentation force.

## Figures and Tables

**Table 1:** Material properties of fused silica glass.

Type of Value	Plane Strain Modulus, E* (MPa)	Poisson’s Ratio, ν(-)
Certified value	75,100 ± 300	0.16
Measured value	74,990	-
SD	1360	-

**Table 2:** Basic equations of brittle phase-field formulation.

Total Energy Functional	
$\Psi =\Psi^{b}+\Psi^{s}=\int_{\Omega /\Gamma }\psi_{e}\left(\varepsilon \right)d\Omega +\int_{\Gamma }G_{c}d\Gamma$	(1)
Elastic deformation energy density	
$\psi_{e}=\frac{1}{2}\lambda tr^{2}\left(\varepsilon \right)+\mu tr\left(\varepsilon^{2}\right)$	(2)
Regularized energy functional	
$\Psi \left(u,\phi \right)=\int_{\Omega }g\left(\phi \right)\psi_{e}\left(\varepsilon \left(u\right)\right)d\Omega +\int_{\Omega }\frac{G_{c}}{2}\left[l{\left(\nabla \phi \right)}^{2}+\frac{1}{l}\phi^{2}\right]d\Omega$	(3)
Governing equations through the principle of virtual work	
$\int_{\Omega }{\left(1-\phi \right)}^{2}\frac{\partial \psi_{e}\left(\varepsilon \right)}{\partial \varepsilon }\delta \varepsilon d\Omega =F^{\mathrm{ext}}\delta u$ $\int_{\Omega }\left\{G_{c}l\Delta \phi \Delta \left(\delta \phi \right)+\frac{G_{c}}{l}\phi \delta \phi \right\}d\Omega =\int_{\Omega }2\left(1-\phi \right)H\left(t\right)\delta \phi d\Omega$ $H\left(t\right):=\max_{\tau =\left[0,t\right]}\psi_{e}\left(\tau \right)$	(4)
Degradation function	
$g\left(\phi \right)={\left(1-\phi \right)}^{2}$	(5)
Crack density function (AT2—Ambrosio-Tortorelli [24,25])	
$\gamma \left(\phi ,\nabla \phi \right)=\frac{1}{2}\left[\frac{1}{l}\phi^{2}+l{\left|\nabla \phi \right|}^{2}\right]$	(6)
Length scale parameter	
$l=\frac{27}{256}\frac{G_{C}E}{{\left(\sigma^{max}\right)}^{2}}$	(7)

**Table 3:** Spectral split energy decomposition main equations, In the table, the well-known formula of the model firstly proposed in [30] are given for completeness.

if $\varepsilon_{1}>0$	
$\psi_{0}^{+}=\frac{Ev}{2\left(1+v\right)\left(1-2v\right)}{\left(\varepsilon_{1}+\varepsilon_{2}+\varepsilon_{3}\right)}^{2}+\frac{E}{2\left(1+v\right)}\left(\varepsilon_{1}^{2}+\varepsilon_{2}^{2}+\varepsilon_{3}^{3}\right),\psi_{0}^{-}=0$	(11)
else if $\varepsilon_{2}+v\varepsilon_{1}>0$	
$\psi_{0}^{+}=\frac{Ev}{2\left(1+v\right)\left(1-2v\right)}{\left(\varepsilon_{3}+\varepsilon_{2}+2v\varepsilon_{1}\right)}^{2}+\frac{E}{2\left(1+v\right)}\left({\left(\varepsilon_{3}+v\varepsilon_{1}\right)}^{2}+{\left(\varepsilon_{2}+v\varepsilon_{1}\right)}^{2}\right),\;\psi_{0}^{-}=\frac{E}{2}\varepsilon_{1}^{2}$	(12)
else if $\left(1-v\right)\varepsilon_{3}+v\left(\varepsilon_{1}+\varepsilon_{2}\right)>0$	
$\psi_{0}^{+}=\frac{E}{2\left(1-v^{2}\right)\left(1-2v\right)}{\left(\left(1-v\right)\varepsilon_{3}+v\varepsilon_{2}+v\varepsilon_{1}\right)}^{2},\;\psi_{0}^{-}=\frac{E}{2\left(1-v^{2}\right)}\left(\varepsilon_{1}^{2}+\varepsilon_{2}^{2}+2v\varepsilon_{1}\varepsilon_{2}\right)$	(13)
else	
$\psi_{0}^{-}=\frac{Ev}{2\left(1+v\right)\left(1-2v\right)}{\left(\varepsilon_{1}+\varepsilon_{2}+\varepsilon_{3}\right)}^{2}+\frac{E}{2\left(1+v\right)}\left(\varepsilon_{1}^{2}+\varepsilon_{2}^{2}+\varepsilon_{3}^{3}\right)$	(14)

**Table 4:** Elastic material parameters for brittle phase-field formulation used in this study.

Modulus of Elasticity, *E,* MPa	Poisson’s Ratio, *ν*	Fracture Toughness/Energy Release Rate, $G_{C},\;N/\mathrm{mm}$	Tensile Strength, $\sigma^{max},\;\mathrm{MPa}$
75,000	0.16	0.006	4000

## Data Availability

Not applicable.
